# Identification of miRNA-target gene regulatory networks in liver fibrosis based on bioinformatics analysis

**DOI:** 10.7717/peerj.11910

**Published:** 2021-08-06

**Authors:** Yang Tai, Chong Zhao, Jinhang Gao, Tian Lan, Huan Tong

**Affiliations:** 1 Laboratory of Gastroenterology and Hepatology, West China Hospital, Sichuan University, Chengdu, China; 2 Department of Gastroenterology, West China Hospital, Sichuan University, Chengdu, China

**Keywords:** miRNA, mRNA, Liver fibrosis, Bioinformatics, Hepatic stellate cell

## Abstract

**Background:**

Liver cirrhosis is one of the leading causes of death worldwide. MicroRNAs (miRNAs) can regulate liver fibrosis, but the underlying mechanisms are not fully understood, and the interactions between miRNAs and mRNAs are not clearly elucidated.

**Methods:**

miRNA and mRNA expression arrays of cirrhotic samples and control samples were acquired from the Gene Expression Omnibus database. miRNA-mRNA integrated analysis, functional enrichment analysis and protein-protein interaction (PPI) network construction were performed to identify differentially expressed miRNAs (DEMs) and mRNAs (DEGs), miRNA-mRNA interaction networks, enriched pathways and hub genes. Finally, the results were validated with *in vitro* cell models.

**Results:**

By bioinformatics analysis, we identified 13 DEMs between cirrhotic samples and control samples. Among these DEMs, six upregulated (hsa-miR-146b-5p, hsa-miR-150-5p, hsa-miR-224-3p, hsa-miR-3135b, hsa-miR-3195, and hsa-miR-4725-3p) and seven downregulated (hsa-miR-1234-3p, hsa-miR-30b-3p, hsa-miR-3162-3p, hsa-miR-548aj-3p, hsa-miR-548x-3p, hsa-miR-548z, and hsa-miR-890) miRNAs were further validated in activated LX2 cells. miRNA-mRNA interaction networks revealed a total of 361 miRNA-mRNA pairs between 13 miRNAs and 245 corresponding target genes. Moreover, PPI network analysis revealed the top 20 hub genes, including *COL1A1*, *FBN1* and *TIMP3,* which were involved in extracellular matrix (ECM) organization; *CCL5*, *CXCL9*, *CXCL12*, *LCK* and *CD24*, which participated in the immune response; and* CDH1*, *PECAM1*, *SELL* and *CAV1,* which regulated cell adhesion. Functional enrichment analysis of all DEGs as well as hub genes showed similar results, as ECM-associated pathways, cell surface interaction and adhesion, and immune response were significantly enriched in both analyses.

**Conclusions:**

We identified 13 differentially expressed miRNAs as potential biomarkers of liver cirrhosis. Moreover, we identified 361 regulatory pairs of miRNA-mRNA and 20 hub genes in liver cirrhosis, most of which were involved in collagen and ECM components, immune response, and cell adhesion. These results would provide novel mechanistic insights into the pathogenesis of liver cirrhosis and identify candidate targets for its treatment.

## Introduction

Liver cirrhosis is a serious public health problem in the world, which causes approximately 1 million human deaths each year (Asrani et al., 2019). The most common primary etiologies for cirrhosis include hepatitis B virus (HBV), hepatitis C virus (HCV), as well as nonalcoholic fatty liver disease (NAFLD) and alcoholic liver disease (ALD) (Asrani et al., 2019). In the context of liver injury and inflammation, excessive amounts of extracellular matrix (ECM) are deposited in the liver and play a central role in the pathogenesis of fibrosis and cirrhosis (Friedman, 2003). This process is regulated by various cells and molecular pathways, such as the activation of hepatic stellate cells (HSCs) and portal fibroblasts (Iwaisako et al., 2014; Nishio et al., 2019), infiltration of immune cells (Gehrke et al., 2018; Krenkel & Tacke, 2017), and dedifferentiation of liver sinusoidal epithelial cells (Ding et al., 2014). However, the mechanisms underlying the development of liver fibrosis are not fully understood. Currently, no treatment can effectively reverse decompensated liver cirrhosis. Therefore, studies further elucidating the pathophysiological mechanisms of the disease are warranted to provide hints for treatment of human liver cirrhosis.

MicroRNAs (miRNAs) are a class of small, noncoding RNAs that can bind to messenger RNAs (mRNAs) and repress gene expression (Ambros, 2004). miRNAs play pivotal roles in regulating diverse biological and pathological processes, including cell proliferation (Shenoy & Blelloch, 2014), metabolism (Agbu & Carthew, 2021), inflammation (Naqvi et al., in press) and disease progression, especially chronic liver diseases (Szabo & Bala, 2013; Wang et al., 2021). miRNAs specifically enriched in the liver can regulate hepatic glucose and lipid metabolism, and their expression is correlated with the disease severity of nonalcoholic steatohepatitis (NASH) (Miyaaki et al., 2014; Yamada et al., 2013). Regarding the effect of miRNAs on liver fibrosis, previous studies have yielded inconsistent results, as some showed that miRNAs were profibrogenic, while others showed that miRNAs exerted antifibrogenic effects. For example, Zhang et al. found that inhibition of miR-21, a miRNA enriched in fibrotic tissues, dramatically reduced HSC activation and ameliorated liver fibrosis by inhibiting the transforming growth factor- *β* (TGF-*β*) pathway (Zhang et al., 2013). Moreover, treatment with miR-29a, the miRNA most highly expressed in HSCs, significantly attenuated liver fibrosis in animal models by downregulating ECM-related gene expression (Matsumoto et al., 2016; Roderburg et al., 2011). In contrast, Caviglia et al. found that neither the specific deletion of miR-21 in HSCs nor the pan-deletion of miRNAs in HSCs exerted significant effects on HSC activation and liver fibrosis (Caviglia et al., 2018). As shown, the regulatory effect of miRNAs on liver fibrosis is still under debate. Moreover, little is known about the downstream targets and biological pathways involved in the regulation of liver fibrosis by miRNAs.

In this study, we analyzed two microarray datasets to identify differentially expressed miRNAs and mRNAs between cirrhotic patients and healthy controls. Moreover, miRNA-mRNA integrated analysis, functional enrichment analysis and protein-protein interaction (PPI) network construction were performed to better understand the downstream effects of miRNA-mRNA regulation. Finally, the results were validated with *in vitro* cell models.

## Materials & Methods

### Microarray data

The miRNA expression profile GSE59492 was obtained from the Gene Expression Omnibus database (GEO, http://www.ncbi.nlm.nih.gov/geo/). miRNA expression arrays were performed on liver tissues from five patients with ALD-induced cirrhosis (ALD-CH), 4 patients with HCV-induced cirrhosis (HCV-CH), 5 patients with NASH-induced cirrhosis (NASH-CH) and six patients with noninjured livers (Normal) based on the GPL16384 platform (Affymetrix Multispecies miRNA-3 Array).

The mRNA microarray data of GSE14323 were also extracted from the GEO database and were generated using an Affymetrix Human Genome U133A Array (GPL96) and Affymetrix Human Genome U133A 2.0 Array (GPL571). A total of 36 liver samples were included in this dataset, 17 of which were from cirrhotic patients (Cirrhosis) whereas 19 were from normal controls (Normal).

### Identification of differentially expressed miRNAs and mRNAs

The raw data were preprocessed by background correction, gene symbol transformation, and normalization through R programming. Then, the Limma R package (v3.38.3) was used to identify the differentially expressed miRNAs (DEMs) and mRNAs (DEGs) between cirrhotic patients and controls with an unpaired *t*-test. With the thresholds of *P* < 0.05 and —fold change— ≥ 1.5, DEMs were identified between ALD-CH and Normal, HCV-CH and Normal, and NASH-CH and Normal. A Venn diagram was generated using the online tool jvenn (http://www.bioinformatics.com.cn/static/others/jvenn/) to overlap the DEMs in ALD-CH, HCV-CH, and NASH-CH. In addition, mRNAs with *P* < 0.05 and —fold change—≥ 2 were selected as the significant DEGs between cirrhotic samples and normal samples. Additionally, volcano plots and heatmaps were generated to visualize the significant gene expression changes using GraphPad Prism 8.4.0 and TB (Toolbox for Biologists) tools software (v1.082) (Chen et al., 2020a), respectively.

### miRNA-mRNA integrated analysis

**Figure 1:**
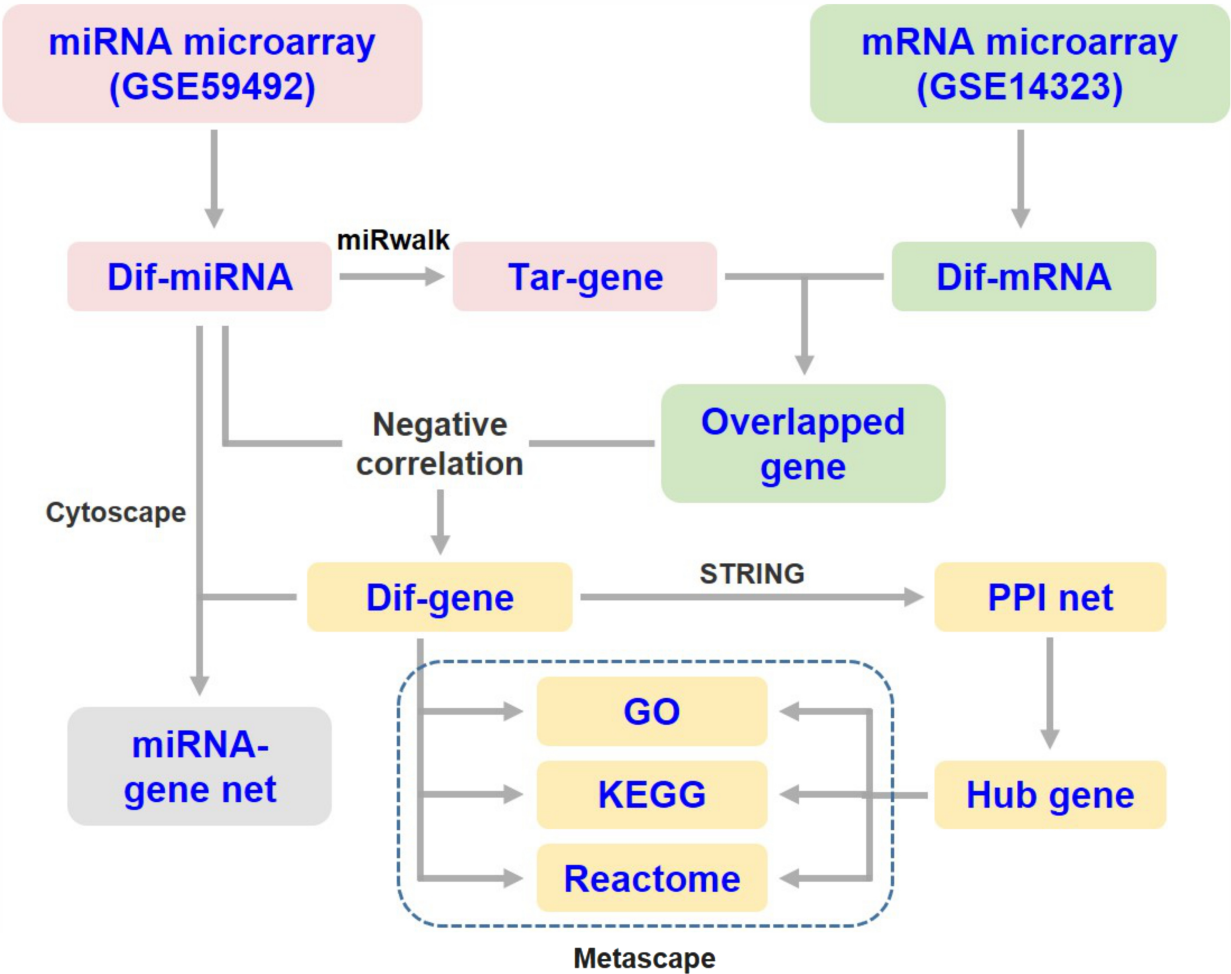
Flow diagram of data collection and processing. Dif-, differentially expressed; PPI, protein-protein interaction; Tar-, target.

The target genes of DEMs were predicted using the online tool miRWalk 3.0 (http://mirwalk.umm.uni-heidelberg.de/) (Sticht et al., 2018). The overlapping genes between the predicted DEM targets and DEGs were selected for integrated analysis. Since miRNAs generally suppress the expression of target mRNAs, only those differentially expressed target genes that were negatively correlated with the specific miRNA were subjected to subsequent analysis (Fig. 1). Then, the miRNA-mRNA interaction networks in liver cirrhosis were constructed and visualized using Cytoscape software (v3.8.2).

### Functional enrichment analysis

Using Metascape (https://metascape.org/) (Zhou et al., 2019), GO analysis was performed to annotate the biological process (BP), cellular component (CC) and molecular function (MF) of the dysregulated genes, and KEGG and Reactome pathway enrichment were used to identify the critical signal pathways of the DEGs. Enrichment analysis was plotted by http://www.bioinformatics.com.cn and https://cloud.oebiotech.cn, two online platforms for data analysis and visualization. Any GO terms and pathways with a *P* value less than 0.05 were considered significantly enriched.

### PPI network construction and hub genes identification

The differentially expressed target genes were mapped to the STRING database (https://string-db.org/) (Szklarczyk et al., 2019) to screen PPIs, and the results were visualized by Cytoscape software as a network structure. Each node represented a protein encoded by a DEG, and the edges between nodes represented interactions between proteins. The cytoHubba plug-in of Cytoscape was used to calculate the degree rank of the hub genes, and the top 20 were identified for further enrichment analysis and PPI subnetwork construction.

### Cell culture and treatment

The human HSC line LX2 was obtained from Procell Life Science and Technology Co., Ltd. (Wuhan, China). The cells were cultured in Dulbecco’s modified Eagle’s medium (DMEM, HyClone, USA) supplemented with 10% fetal bovine serum (FBS, Biological Industries, USA), and 100 U/mL penicillin and 100 µg/mL streptomycin (HyClone) in a humidified atmosphere at 37 °C with 5% CO_2_ in air. After 16 h of serum starvation, LX2 cells were treated with 5 ng/mL TGF-*β*1 (PeproTech, USA) for 24 h and then harvested for gene and protein analysis.

### Immunofluorescence staining

LX2 cells cultured on coverslips at the bottom of 24-well plates were fixed with 4% PFA before permeabilization with 0.1% Triton X-100. After blocking with 10% goat serum, the cells were incubated with anti *α*-SMA (1:200, Cat# NB600-536, Novus Biologicals, USA) overnight at 4 °C followed by incubation with TRITC-conjugated secondary antibodies (1:200, Cat# ZF-0313, ZSGB-BIO, Beijing, China) at room temperature for 30 min. The cell nuclei were then stained with 4′,6-diamino-2-phenylindole (DAPI, Roche, Switzerland). Coverslips were mounted with anti-fading medium and visualized under a fluorescence microscope (Olympus, Japan).

### Western blotting

Whole proteins from cells were extracted using a protein extraction kit (Nanjing KeyGen Biotech Co., Ltd, Nanjing, China). SDS-PAGE (10%) was used to separate equal amounts of protein from each sample, and then samples were transferred to PVDF membranes (Merck Millipore, USA). The membranes were blocked with 5% nonfat milk and incubated with anti *α*-SMA (1:1000, Cat# NB600-536, Novus Biologicals) and anti-GAPDH (1:2000, Cat# sc-166574, Santa Cruz Biotechnology, USA) antibodies overnight at 4 °C. After the PVDF membranes were washed and incubated with HRP-conjugated secondary antibodies (1:20000, Cat# ZB-2305, ZSGB-BIO) at room temperature for 2 h, the protein bands were visualized by chemiluminescence using Western Blotting Luminol Reagent (Santa Cruz Biotechnology), and densitometric analyses were performed using Quantity One software (v4.6.2). The protein levels were normalized against GAPDH and were shown as fold changes relative to the control group.

### Quantitative real-time PCR (qRT-PCR)

Total RNA was extracted from the cell lysates using a miRNA Isolation Kit (Foregene, Chengdu, China) according to the manufacturer’s instructions. Then, equal amounts of 1 µg RNA were reverse transcribed into cDNA using the miRcute Plus miRNA First-Strand cDNA Synthesis Kit (Tiangen, Beijing, China). qRT-PCR was performed in triplicate using SYBR Green qPCR Master Mix (Bimake, USA) on the CFX96 Real-Time PCR Detection System (Bio-Rad). The relative miRNA expression levels were normalized to the U6 snRNA expression level using the 2^−ΔΔCT^ method, and the results were shown as fold changes relative to the control group. The primer sequences were listed in Table 1.

**Table 1:** Primer sequences for qRT-PCR.

**miRNA symbol**	**Forward sequence (5′–3′)**
hsa-miR-1234-3p	TCGGCCTGACCACCCACCCCAC
hsa-miR-146b-5p	TGAGAACTGAATTCCATAGGCT
hsa-miR-150-5p	CTGGTACAGGCCTGGGGGACAG
hsa-miR-224-3p	AAAATGGTGCCCTAGTGACTACA
hsa-miR-30b-3p	CTGGGAGGTGGATGTTTACTTC
hsa-miR-3135b	GGCTGGAGCGAGTGCAGTGGTG
hsa-miR-3162-3p	TCCCTACCCCTCCACTCCCCA
hsa-miR-3195	CGCGCCGGGCCCGGGTT
hsa-miR-4725-3p	TGGGGAAGGCGTCAGTGTCGGG
hsa-miR-548aj-3p	TAAAAACTGCAATTACTTTTA
hsa-miR-548x-3p	TAAAAACTGCAATTACTTTC
hsa-miR-548z	CAAAAACCGCAATTACTTTTGCA
hsa-miR-890	TACTTGGAAAGGCATCAGTTG

### Statistical analysis

All data were expressed as mean ± standard deviation and were analyzed using SPSS 19.0 software (SPSS, USA). Student’s *t*-test was performed for comparisons between two groups, and a *P* value < 0.05 was considered statistically significant.

## Results

### Identification of DEMs and DEGs

**Figure 2:**
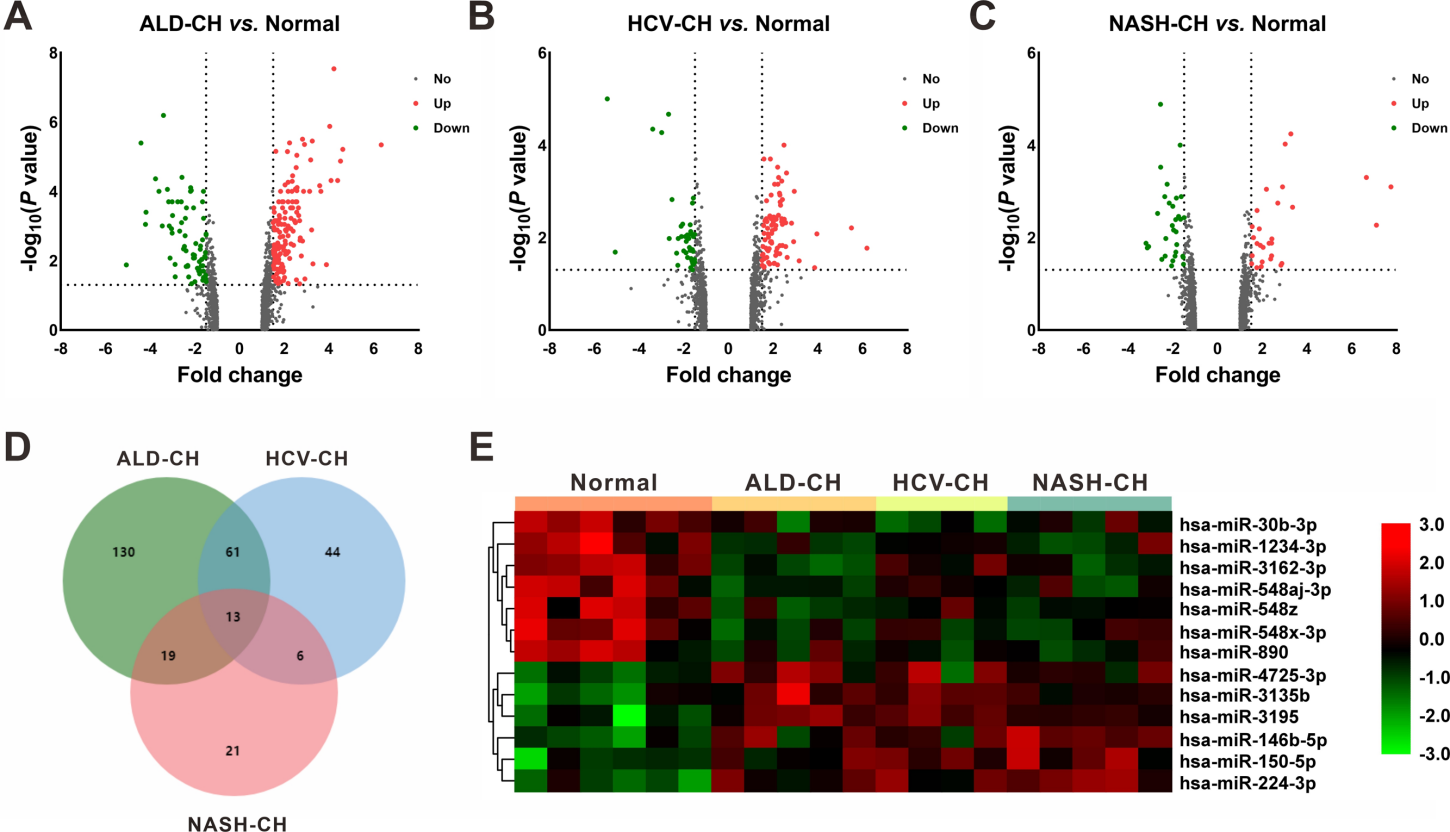
Identification of differentially expressed miRNAs (DEMs). (A–C): Volcano plots of differentially expressed miRNAs between ALD-CH and Normal (A), HCV-CH and Normal (B), and NASH-CH and Normal (C), respectively. The red dots represented upregulated miRNAs, the green dots represented downregulated miRNAs, and the gray blots represented miRNAs with no significant difference. D: Venn diagram of identified DEMs among ALD-CH, HCV-CH and NASH-CH. E: Heatmap of DEMs *via* hierarchical cluster analysis. Colors from green to red represented the miRNA expression abundance from poor to rich. ALD-CH, alcohol liver disease-induced cirrhosis; DEMs, differentially expressed miRNAs; HCV-CH, hepatitis C virus-induced cirrhosis; NASH-CH, nonalcoholic steatohepatitis-induced cirrhosis.

In the GSE59492 profile, 223, 124, and 59 DEMs with *P* < 0.05 and —fold change— ≥ 1.5 were identified by comparing ALD-CH and Normal, HCV-CH and Normal, and NASH-CH and Normal, respectively (Figs. 2A–2C). The candidate DEMs generated by these comparisons were intersected using a Venn diagram (Fig. 2D). As shown in Fig. 2E, a total of 13 DEMs were identified in cirrhotic livers, of which 6 were upregulated (hsa-miR-146b-5p, hsa-miR-150-5p, hsa-miR-224-3p, hsa-miR-3135b, hsa-miR-3195 and hsa-miR-4725-3p) and 7 were downregulated (hsa-miR-1234-3p, hsa-miR-30b-3p, hsa-miR-3162-3p, hsa-miR-548aj-3p, hsa-miR-548x-3p, hsa-miR-548z and hsa-miR-890). Detailed information pertaining to these DEMs was listed in Table 2.

**Table 2:** The differentially expressed miRNAs.

**miRNA symbol**	**Up/Down**	**ALD-CH*vs.*Normal**		**HCV-CH*vs.*Normal**		**NASH-CH*vs.*Normal**	
		**Fold change**	***P*value**	**Fold change**	***P*value**	**Fold change**	***P*value**
hsa-miR-1234-3p	Down	−2.43	6.00E−04	−1.83	9.80E−03	−2.37	1.30E−03
hsa-miR-146b-5p	Up	2.41	3.60E−03	2.18	1.69E−02	3.02	9.45E−05
hsa-miR-150-5p	Up	2.9	4.42E−06	2.49	4.00E−03	2.9	8.00E−04
hsa-miR-224-3p	Up	2.04	2.70E−03	2.41	1.50E−03	3.27	5.70E−05
hsa-miR-30b-3p	Down	−1.66	2.50E−03	−2.99	5.33E−05	−1.88	4.10E−03
hsa-miR-3135b	Up	2.28	3.00E−04	2.38	6.00E−04	1.76	1.35E−02
hsa-miR-3162-3p	Down	−3.41	6.41E−07	−1.7	8.40E−03	−2.56	1.31E−05
hsa-miR-3195	Up	2.56	9.59E−05	2.48	1.00E−04	1.76	2.60E−03
hsa-miR-4725-3p	Up	2.81	2.70E−03	2.56	1.57E−02	1.75	4.49E−02
hsa-miR-548aj-3p	Down	−2.2	1.00E−04	−1.75	1.64E−02	−2.26	7.00E−04
hsa-miR-548x-3p	Down	−2.06	3.00E−04	−1.57	9.50E−03	−1.66	4.00E−03
hsa-miR-548z	Down	−2.38	3.00E−04	−1.89	1.21E−02	−1.85	1.40E−03
hsa-miR-890	Down	−2.07	8.40E−03	−1.84	8.80E−03	−1.97	6.90E−03

**Notes.**

ALD-CH, alcohol liver disease-induced cirrhosis; HCV-CH, hepatitis C virus-induced cirrhosis; NASH-CH, non-alcoholic steatohepatitis induced cirrhosis.

**Figure 3:**
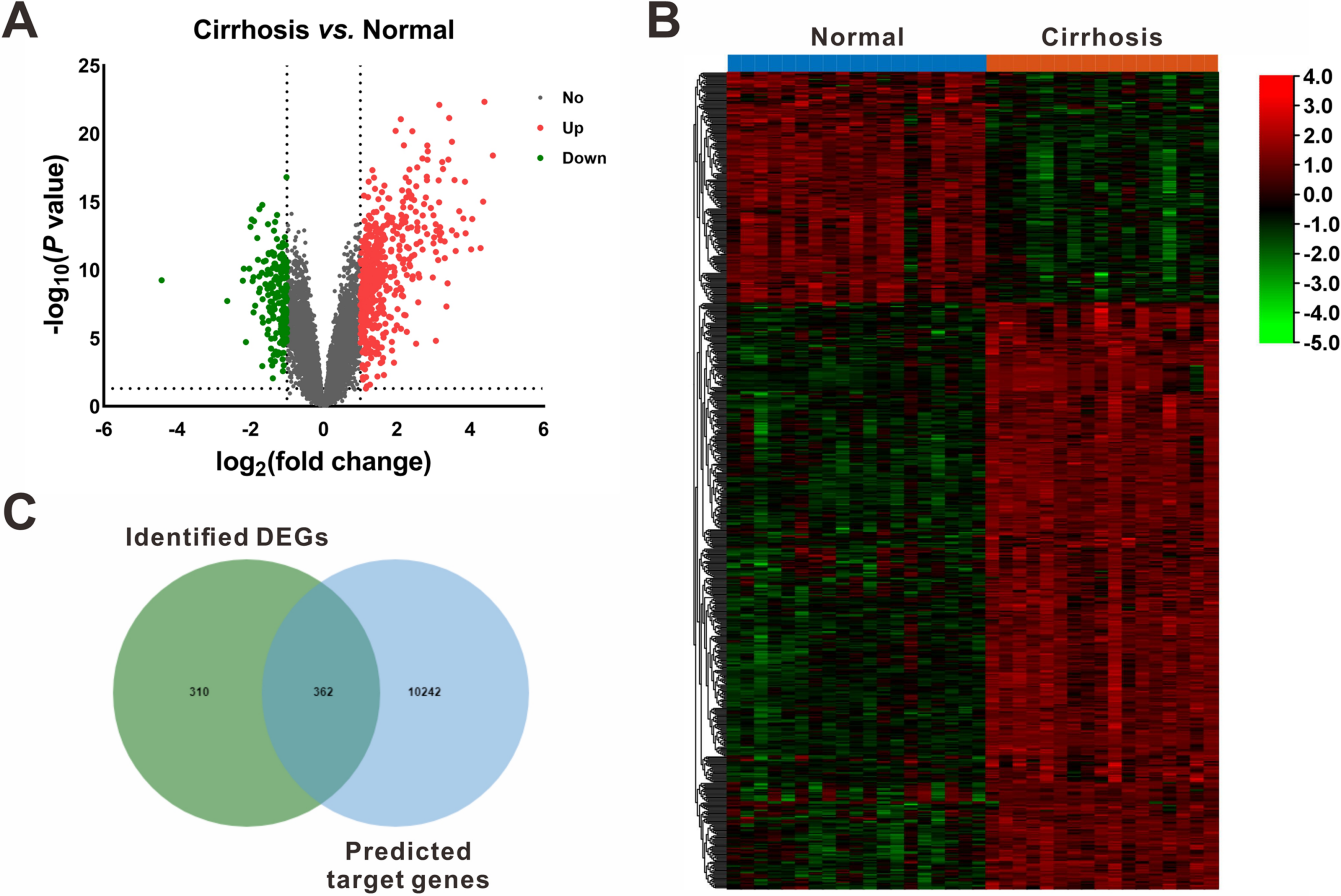
Identification of differentially expressed mRNAs (DEGs). (A) Volcano plot of differentially expressed mRNAs between Cirrhosis and Normal. The red dots represented upregulated mRNAs, the green dots represented downregulated mRNAs, and the gray blots represented mRNAs with no significant difference. (B) Heatmap of DEGs *via* hierarchical cluster analysis. Colors from green to red represented the mRNAs expression abundance from poor to rich. (C) Venn diagram of the identified DEGs and the predicted target genes of DEMs. DEGs, differentially expressed mRNAs; DEMs, differentially expressed miRNAs.

On the other hand, based on the screening criteria of *P* < 0.05 and —fold change— ≥ 2, 672 mRNAs (482 upregulated and 190 downregulated) were shown to be differentially expressed between cirrhotic samples and control samples in the GSE14323 dataset (Fig. 3A). The volcano plots and heatmap of the DEGs were shown in Figs. 3A and 3B, respectively.

### miRNA-mRNA interaction networks

**Figure 4:**
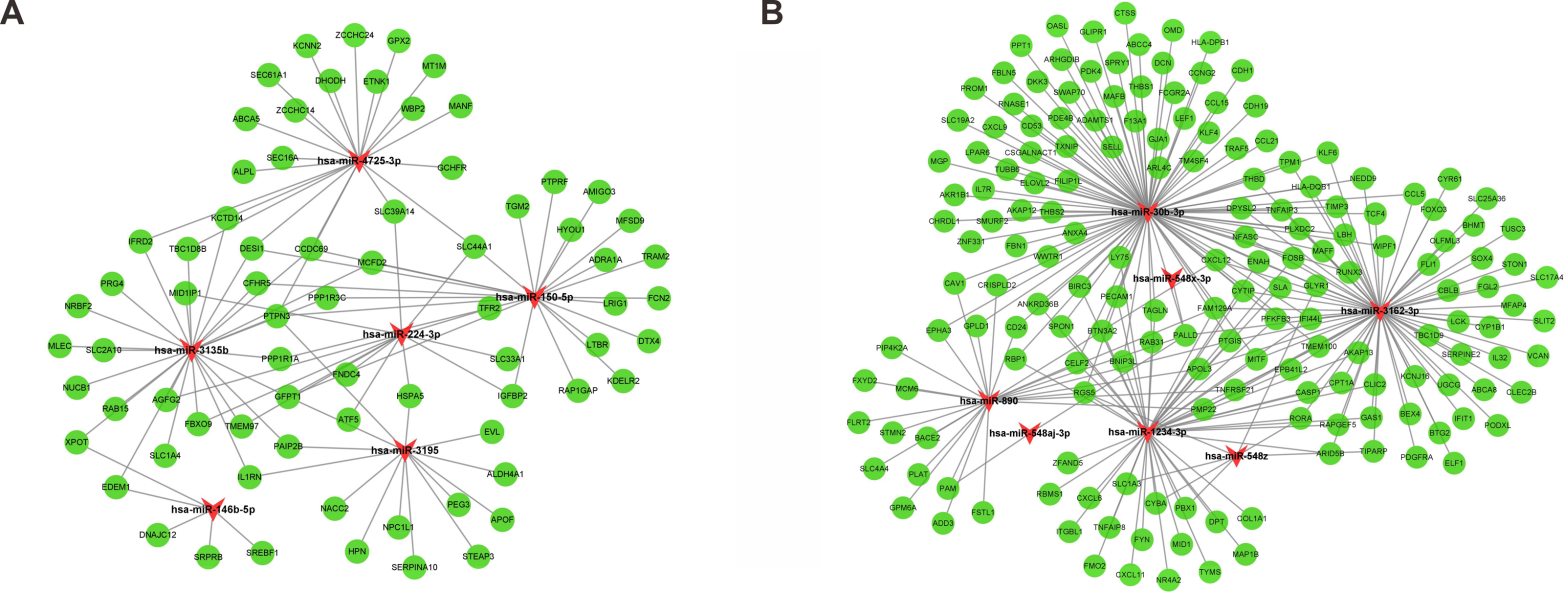
MiRNA-mRNA interaction networks. The miRNA-mRNA interaction networks for upregulated miRNAs *vs.* downregulated mRNAs (A), and downregulated miRNAs *vs.* upregulated mRNAs (B). The red arrowheads represented miRNAs, and the green circles represented the target mRNAs.

Following gene expression analysis, 10,604 potential target genes of DEMs were predicted using miRWalk. By considering the overlapping genes, we identified 362 overlapping genes between the predicted target genes and DEGs, which were used for further correlation analysis (Fig. 3C). After filtering out those pairs in which miRNA and mRNA expression shared the same change trend, a total of 361 miRNA-mRNA pairs between 13 miRNAs and 245 corresponding target genes were identified. In these negatively correlating interactions visualized with Cytoscape, 103 regulatory pairs were composed of 6 upregulated miRNAs and 73 downregulated mRNAs (Fig. 4A, Table 3), and 258 pairs were composed of 7 downregulated miRNAs and 172 upregulated mRNAs (Fig. 4B, Table 3). Notably, a single miRNA might interact with different genes, while one gene might also become the shared target of different miRNAs. Except for hsa-miR-548x-3p, the other DEMs were all predicted to interact with multiple target genes. Additionally, *CYTIP*, *ENAH*, *FAM129A*, *FOSB*, *IFI44L*, *MITF*, *PFKFB3*, *PTGIS*, *SLA* and *TMEM100* were identified as targets of hsa-miR-1234-3p, hsa-miR-30b-3p and hsa-miR-3162-3p. These findings revealed a complex regulatory network between miRNAs and mRNAs in the process of liver cirrhosis.

**Table 3:** The target genes for differentially expressed miRNAs.

**miRNA symbol**	**Up/Down**	**Count**	**Target gene**
hsa-miR-1234-3p	Down	50	*AKAP13, ANKRD36B, APOL3, ARID5B, BIRC3, BNIP3L, BTN3A2, CASP1, CELF2, CLIC2, COL1A1, CPT1A, CXCL11, CXCL6, CYBA, CYTIP, DPT, ENAH, EPB41L2, FAM129A, FMO2, FOSB, FYN, GAS1, IFI44L, ITGBL1, LY75, MAP1B, MID1, MITF, NR4A2, PBX1, PECAM1, PFKFB3, PMP22, PTGIS, RAB31, RAPGEF5, RBMS1, RBP1, RGS5, RORA, SLA, SLC1A3, SPON1, TAGLN, TMEM100, TNFAIP8, TYMS, ZFAND5*
hsa-miR-146b-5p	Up	5	*DNAJC12, EDEM1, SREBF1, SRPRB, XPOT*
hsa-miR-150-5p	Up	23	*ADRA1A, AMIGO3, CCDC69, CFHR5, DESI1, DTX4, FCN2, GFPT1, HYOU1, IGFBP2, KDELR2, LRIG1, LTBR, MCFD2, MFSD9, PPP1R1A, PPP1R3C, PTPN3, PTPRF, RAP1GAP, SLC44A1, TGM2, TRAM2*
hsa-miR-224-3p	Up	13	*AGFG2, ATF5, FBXO9, FNDC4, GFPT1, HSPA5, IGFBP2, MID1IP1, SLC33A1, SLC39A14, SLC44A1, TFR2, TMEM97*
hsa-miR-30b-3p	Down	100	*ABCC4, ADAMTS1, AKAP12, AKR1B1, ANKRD36B, ANXA4, ARHGDIB, ARL4C, BIRC3, BNIP3L, BTN3A2, CAV1, CCL15, CCL21, CCL5, CCNG2, CD24, CD53, CDH1, CDH19, CELF2, CHRDL1, CRISPLD2, CSGALNACT1, CTSS, CXCL12, CXCL9, CYTIP, DCN, DKK3, DPYSL2, ELOVL2, ENAH, EPHA3, F13A1, FAM129A, FBLN5, FBN1, FCGR2A, FILIP1L, FOSB, GJA1, GLIPR1, GLYR1, GPLD1, HLA-DPB1, HLA-DQB1, IFI44L, IL7R, KLF4, KLF6, LBH, LEF1, LPAR6, LY75, MAFB, MAFF, MGP, MITF, NEDD9, NFASC, OASL, OMD, PALLD, PDE4B, PDK4, PECAM1, PFKFB3, PLXDC2, PPT1, PROM1, PTGIS, RAB31, RBP1, RGS5, RNASE1, RUNX3, SELL, SLA, SLC19A2, SMURF2, SPON1, SPRY1, SWAP70, TAGLN, TCF4, THBD, THBS1, THBS2, TIMP3, TM4SF4, TMEM100, TNFAIP3, TPM1, TRAF5, TUBB6, TXNIP, WIPF1, WWTR1, ZNF331*
hsa-miR-3135b	Up	26	*AGFG2, CCDC69, CFHR5, DESI1, EDEM1, FBXO9, GFPT1, IFRD2, IL1RN, KCTD14, MCFD2, MID1IP1, MLEC, NRBF2, NUCB1, PAIP2B, PPP1R1A, PPP1R3C, PRG4, PTPN3, RAB15, SLC1A4, SLC2A10, TBC1D8B, TMEM97, XPOT*
hsa-miR-3162-3p	Down	72	*ABCA8, AKAP13, APOL3, ARID5B, BEX4, BHMT, BTG2, CASP1, CBLB, CCL5, CLEC2B, CLIC2, CPT1A, CXCL12, CYP1B1, CYR61, CYTIP, DPYSL2, ELF1, ENAH, EPB41L2, FAM129A, FGL2, FLI1, FOSB, FOXO3, GAS1, GLYR1, HLA-DQB1, IFI44L, IFIT1, IL32, KCNJ16, KLF6, LBH, LCK, MAFF, MFAP4, MITF, NEDD9, NFASC, OLFML3, PALLD, PDGFRA, PFKFB3, PLXDC2, PMP22, PODXL, PTGIS, RAPGEF5, RORA, RUNX3, SERPINE2, SLA, SLC17A4, SLC25A36, SLIT2, SOX4, STON1, TBC1D9, TCF4, THBD, TIMP3, TIPARP, TMEM100, TNFAIP3, TNFRSF21, TPM1, TUSC3, UGCG, VCAN, WIPF1*
hsa-miR-3195	Up	14	*ALDH4A1, APOF, EVL, GFPT1, HPN, HSPA5, IL1RN, NACC2, NPC1L1, PAIP2B, PEG3, PTPN3, SERPINA10, STEAP3*
hsa-miR-4725-3p	Up	22	*ABCA5, ALPL, CCDC69, DESI1, DHODH, ETNK1, GCHFR, GPX2, IFRD2, KCNN2, KCTD14, MANF, MT1M, PTPN3, SEC16A, SEC61A1, SLC39A14, SLC44A1, TBC1D8B, WBP2, ZCCHC14, ZCCHC24*
hsa-miR-548aj-3p	Down	2	*PALLD, PAM*
hsa-miR-548x-3p	Down	1	*PALLD*
hsa-miR-548z	Down	7	*CYBA, GLYR1, PBX1, RGS5, RORA, SLC1A3, TIPARP*
hsa-miR-890	Down	26	*ADD3, APOL3, BACE2, CAV1, CD24, CELF2, CRISPLD2, CXCL12, EPHA3, FAM129A, FLRT2, FSTL1, FXYD2, GPLD1, GPM6A, MCM6, PALLD, PAM, PIP4K2A, PLAT, PMP22, PTGIS, RGS5, SLC4A4, STMN2, TNFRSF21*

### GO enrichment and pathway analyses

**Figure 5:**
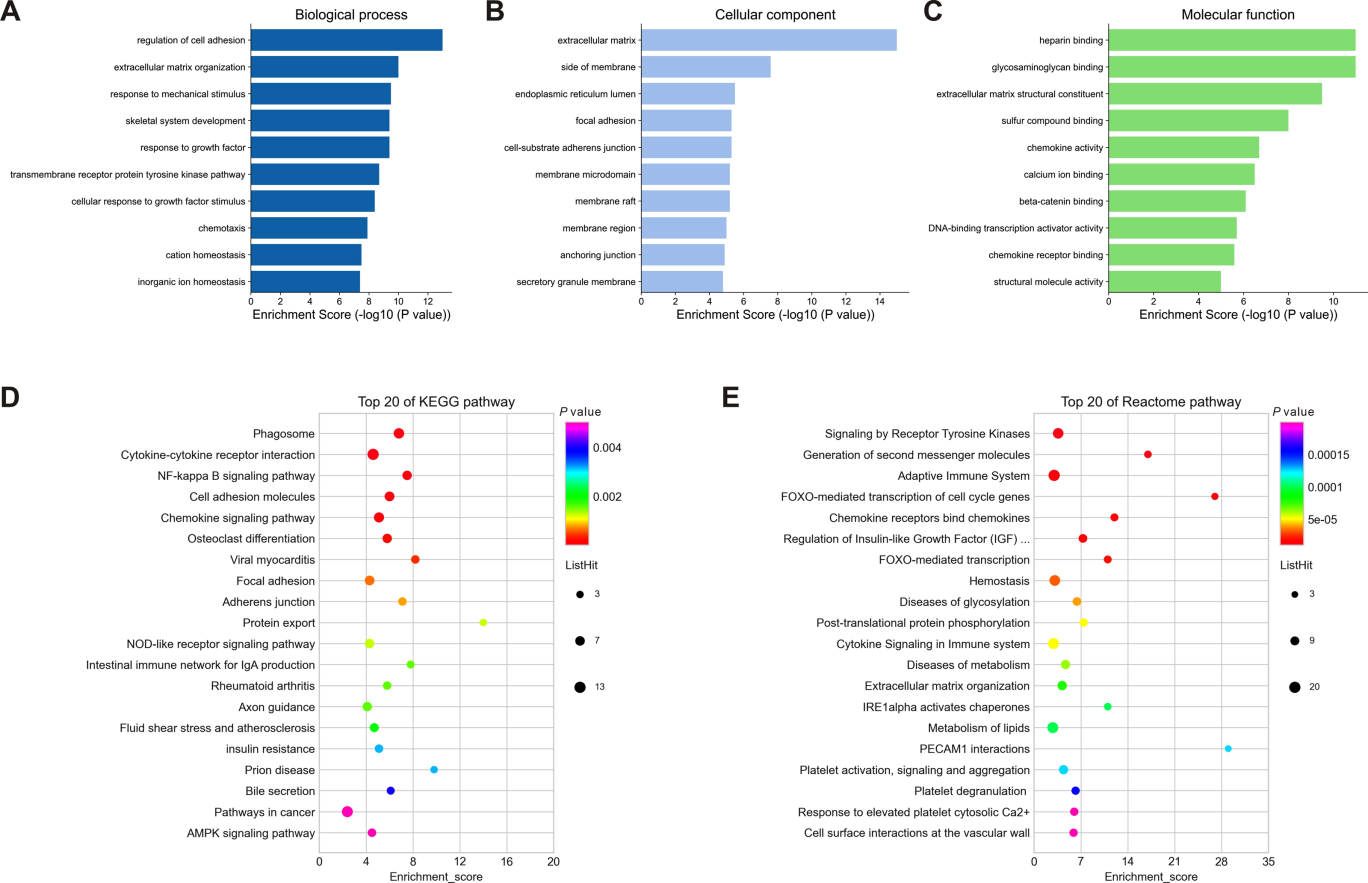
Enrichment analysis of differentially expressed mRNAs (DEGs). (A–C): Top 10 enriched GO terms of the corresponding biological process (A), molecular function (B) and cellular component (C) for DEGs. (D–E): Top 20 enriched pathways for DEGs based on the KEGG (D) and Reactome (E) pathway databases. DEGs, differentially expressed mRNAs.

To understand the biological function and key pathways of the DEGs, GO annotation, KEGG pathway and Reactome pathway analyses of 245 dysregulated genes from negative regulatory interactions were performed utilizing the Metascape online tool. The top 10 significantly enriched GO terms, including BP, CC and MF, were presented in Figs. 5A–5C. The significantly enriched entries for BP were regulation of cell adhesion, ECM organization and response to mechanical stimulus (Fig. 5A). Moreover, the ECM, side of membrane and endoplasmic reticulum lumen accounted for the majority of the CC terms (Fig. 5B). The most enriched MFs were functions in heparin binding, glycosaminoglycan binding and ECM structural constituent (Fig. 5C). Moreover, the DEGs were also mapped to the KEGG and Reactome pathway databases. As shown in Figs. 5D–5E, immune response, protein and lipid metabolism, ECM organization, cell surface interaction, and signal transduction were among the top 20 most statistically significant enriched pathways.

### PPI network and hub genes analyses

**Figure 6:**
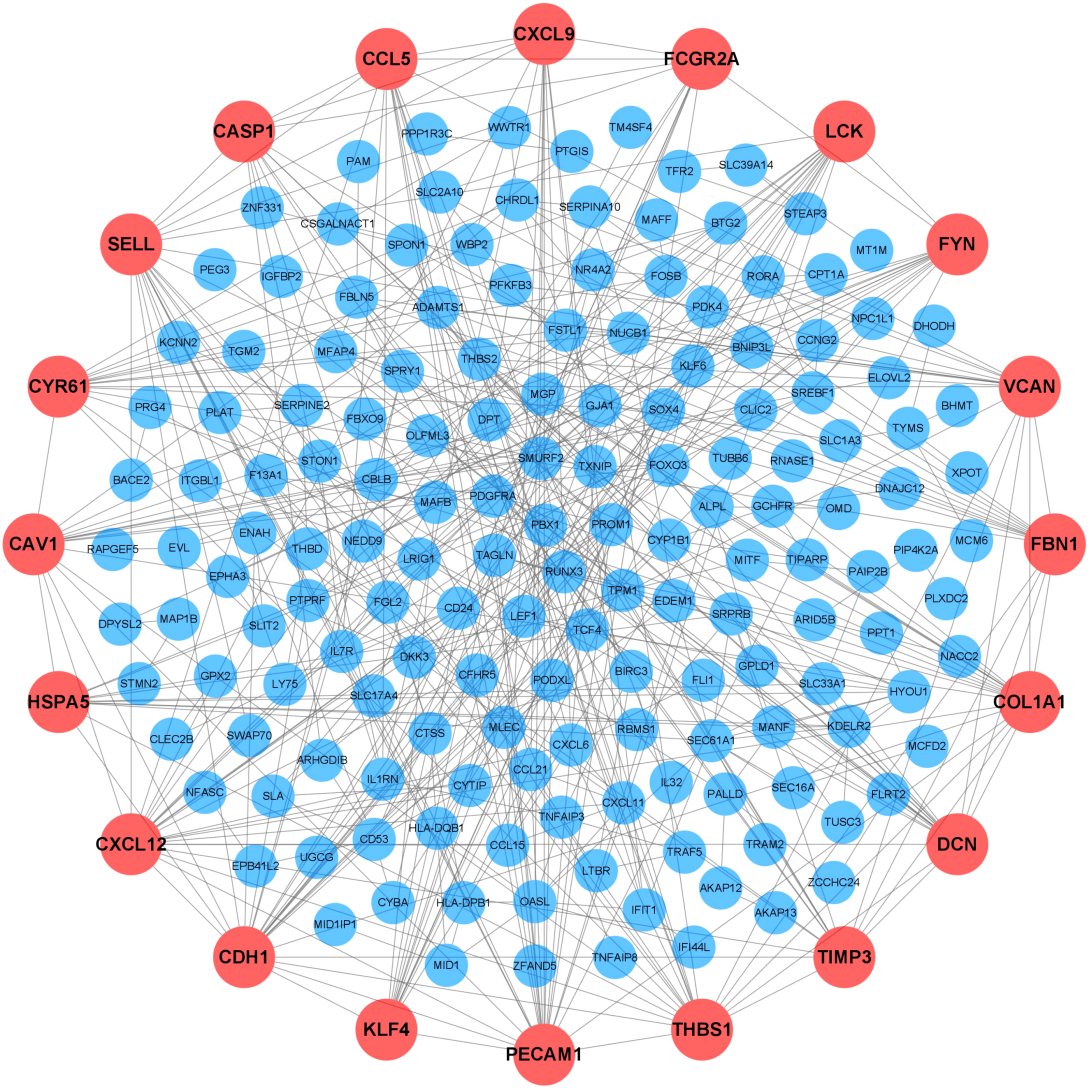
Protein-protein interaction (PPI) network. Each node represented a protein encoded by a DEG, and the edges between nodes represented interactions between proteins. The top 20 hub genes were labeled in orange and associated with larger circles. DEGs, differentially expressed mRNAs; PPI, protein-protein interaction.

**Figure 7:**
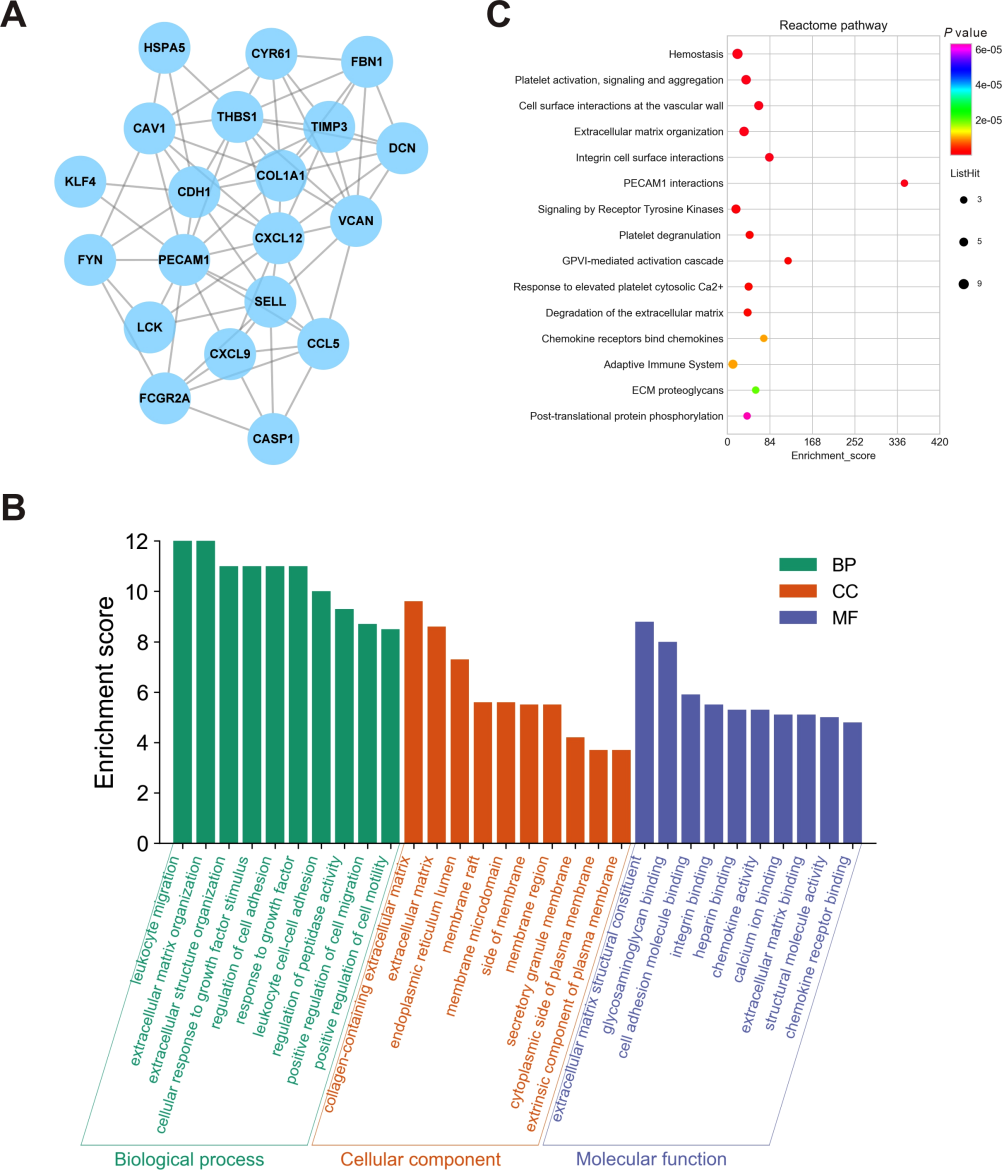
Biological analyses of the hub genes. (A) PPI network analysis of the hub genes. Each node represented a protein encoded by a hub gene, and the edges between nodes represented interactions between proteins. (B) GO enrichment analysis was performed for the hub genes, and the top 10 enriched items were presented in the histogram. (C) Bubble chart of the enriched Reactome pathways for the hub genes. DEGs, differentially expressed mRNAs; PPI, protein-protein interaction.

To further elucidate the interactions among 245 DEGs, we performed PPI network analysis using logical data originating from the STRING database. After removing the unconnected nodes, a PPI network consisting of 180 nodes and 482 edges was constructed and visualized by Cytoscape (Fig. 6). The top 20 hub genes, which were defined as genes that played essential roles in the network, were distinguished according to the degree calculated by cytoHubba of Cytoscape (Table 4). The PPI subnetwork analysis revealed an average combined score of 0.64, indicating medium-high-degree interactions between these hub genes (low confidence: scores < 0.4; medium: 0.4 to 0.7; high: > 0.7, Fig. 7A) (Von Mering et al., 2005). Among the 20 hub genes, *COL1A1*, *FBN1*, and *TIMP3* were involved in ECM organization, *CCL5*, *CXCL9*, *CXCL12*, *LCK* and *CD24* participated in the immune response, and *CDH1*, *PECAM1*, *SELL* and *CAV1* regulated cell adhesion. In support of this, GO annotation analysis showed that hub genes were closely associated with leukocyte migration, ECM, and cell adhesion molecule binding (Fig. 7B). Additionally, pathway enrichment analysis was also performed based on the Reactome pathway database. In total, 15 Reactome pathways, involving ECM organization, chemokines, cell surface interactions and interleukin signaling, were significantly enriched (Fig. 7C).

**Table 4:** Top 20 hub genes in PPI network.

**Rank**	**mRNA symbol**	**Score**	**Rank**	**mRNA symbol**	**Score**
1	*CDH1*	24	11	*CCL5*	15
2	*CXCL12*	23	12	*FYN*	15
3	*PECAM1*	23	13	*FBN1*	15
4	*SELL*	20	14	*CYR61*	15
5	*THBS1*	20	15	*TIMP3*	15
6	*DCN*	19	16	*KLF4*	14
7	*COL1A1*	19	17	*CXCL9*	13
8	*VCAN*	17	18	*CASP1*	13
9	*LCK*	16	19	*CD24*	12
10	*CAV1*	16	20	*HSPA5*	12

**Notes.**

PPI, protein-protein interaction.

### Validation in HSCs by qRT-PCR

**Figure 8:**
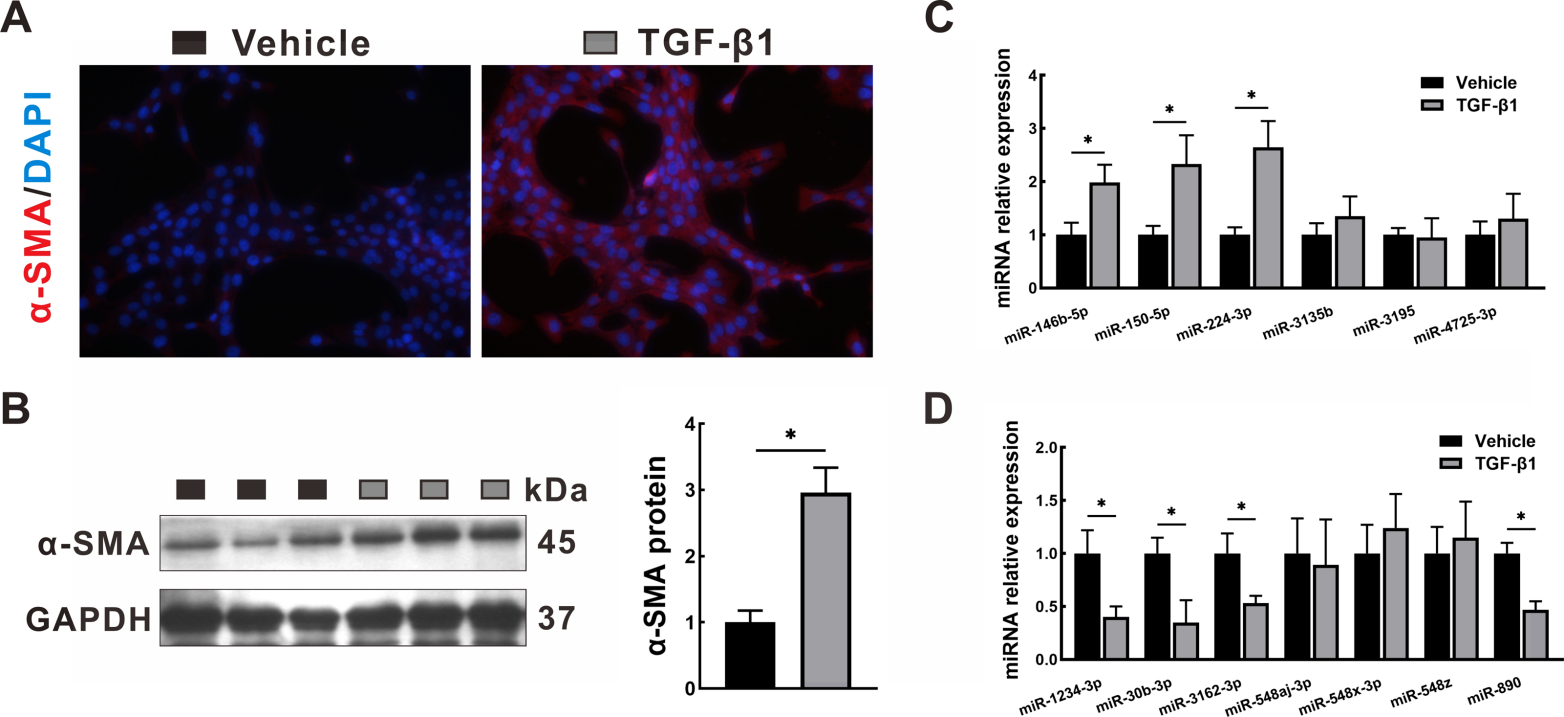
Validation in HSCs by qRT-PCR. LX2 cells were treated with TGF- *β*1 or vehicle alone for 24 h. (A–B) Expression of *α*-SMA was determined by immunofluorescence staining (A, 400 × magnification) and Western blotting (B). (C–D): Expression of upregulated DEMs (C) and downregulated DEMs (D) was validated by qRT-PCR. The data were presented as mean ± standard deviation. *n* = 3/group, * *P* < 0.05. *α*-SMA, *α*-smooth muscle actin; DAPI, 4′, 6-diamino-2-phenylindole; DEMs, differentially expressed miRNAs; GAPDH, glyceraldehyde-3-phosphate dehydrogenase; HSCs, hepatic stellate cells; qRT-PCR, quantitative real-time PCR; TGF- *β*1, transforming growth factor- *β*1.

To validate the transcriptome data, the miRNA expression levels of the identified DEMs were quantified by qRT-PCR in LX2 cells treated with TGF-*β*1, which is a classical cellular model for the study of liver fibrosis (Tsuchida & Friedman, 2017). The activation of LX2 cells was confirmed by the overexpression of *α*-SMA, as proven by immunofluorescence staining (Fig. 8A) and Western blotting (Fig. 8B). After TGF-*β*1 treatment, hsa-miR-146b-5p, hsa-miR-150-5p and hsa-miR-224-3p expression was upregulated, while hsa-miR-1234-3p, hsa-miR-30b-3p, hsa-miR-3162-3p and hsa-miR-890 expression was downregulated. However, no statistically significant difference was observed in the expression of hsa-miR-3135b, hsa-miR-3195, hsa-miR-4725-3p, hsa-miR-548aj-3p, hsa-miR-548x-3p and hsa-miR-548z in the TGF-*β*1 group compared with the control group.

## Discussion

In this study, we identified 13 differentially expressed miRNAs by bioinformatics analysis. Seven of these miRNAs were further confirmed by *in vitro* experiments. In addition, 361 pairs of miRNA-mRNA interactions and 20 hub genes among 245 differentially expressed genes were identified, with multiple pathways involved.

Among these miRNAs, only some have been studied in organ fibrosis. miR-146b-5p has been mainly focused on the heart, and it was found to activate fibroblast proliferation, migration and fibroblast-to-myofibroblast transition, and its silencing could improve cardiac remodeling (Liao et al., 2021). miR-3135b was found to be elevated in hypertrophic scars (Zhang et al., 2020). In addition, miR-150-5p expression was generally positively correlated with fibrosis severity in rats with carbon tetrachloride-induced liver fibrosis (Chen et al., 2020b). On the other hand, in chronic HBV-infected patients, miR-3162-3p expression was higher in the serum of individuals with significant liver fibrosis than those with nonsignificant liver fibrosis (Zhang et al., 2021). In addition to these aforementioned miRNAs and somewhat elusive information, 11 out of 13 miRNAs were shown to be involved in liver cirrhosis for the first time with bioinformatics analysis, and the expression of 7 miRNAs was proved to undergo significant changes in LX2 cells challenged with TGF-*β*1 *in vitro*. Moreover, it was also concluded in this study that miR-150-5p expression was elevated in both cirrhotic tissues and activated LX2 cells, adding more evidence to the debate about whether miR-150-5p expression increases during liver fibrosis (Cabantous et al., 2017; Cai et al., 2018; Chen et al., 2020b).

Liver cirrhosis is the end stage of fibrosis, and ECM and collagen are excessively produced and accumulated in the liver throughout fibrosis (Kisseleva & Brenner, 2021). Our GO enrichment, pathway and hub gene analyses undoubtedly showed that these composition shifts were indispensable in liver cirrhosis. Liver injury is the initiating step of liver cirrhosis, and resident macrophages can detect the injury and then release proinflammatory factors to attract other immune cells (Guillot & Tacke, 2019). In addition, components of the ECM, such as hyaluronan, are implicated in immune regulation. The secretion of hyaluronan has been closely associated with maintaining the myofibroblast phenotype (Meran et al., 2007), and high molecular weight hyaluronan promotes immune tolerance by enhancing the numbers of CD4^+^CD25^+^ regulatory T cells (Bollyky et al., 2009). The most significantly enriched pathway in Reactome pathway analysis, signaling by receptor tyrosine kinases, was also recently reported to be associated with liver fibrosis. Fibroblast growth factor receptor, a major family of receptor tyrosine kinases, plays a key role in the progression and resolution of liver fibrosis by interacting with HSCs, hepatocytes and immune cells (Seitz & Hellerbrand, 2021). In addition, knockdown of *Gab1*, an important protein in the receptor tyrosine kinase pathway, significantly exacerbated liver fibrosis in animal models (Mizutani et al., 2021). According to KEGG pathway analysis, phagosome was most highly enriched pathway, which was consistent with other studies that revealed that autophagy and LC3-associated phagocytosis were closely related to the pathogenesis of liver fibrosis (Li et al., 2020; Wan et al., 2020).

In this study, 20 hub genes in the PPI network were identified. Among them, *CCL5*, *CXCL9*, *CXCL12*, *LCK* and *CD24*, which are thought to participate in the immune response, were identified as the most affected genes, suggesting a disruption of the immune response in liver cirrhosis. Local inflammation in the liver could cause immune cells to migrate to the foci with the assistance of cell adhesion molecules. Moreover, activated myofibroblastic HSCs gradually transform their surrounding ECM into a high-density matrix with increased stiffness; therefore, cell–matrix interactions would be adapted to these changes (Hintermann & Christen, 2019). Our results showed that the expression of cell adhesion molecules, such as *CDH1*, *PECAM1*, *SELL* and *CAV1*, which are canonical molecules in cell adhesion, was evidently changed in liver cirrhosis. Of note, platelet-associated pathways were significantly enriched in Reactome pathway analysis, coinciding with former studies that showed that platelet-derived TGF-*β* is closely involved in the pathogenesis of liver fibrosis (Ahamed & Laurence, 2017; Karolczak & Watala, 2021). Liver sinusoidal epithelial cells can help internalize platelets, thus triggering the profibrogenic effect of platelets (Poisson et al., 2017), which was also reflected in Reactome analysis, as cell surface interactions at the vascular wall was enriched.

*In vitro* cell experiments showed that hsa-miR-146b-5p, hsa-miR-150-5p and hsa-miR-224-3p were upregulated, while hsa-miR-1234-3p, hsa-miR-30b-3p, hsa-miR-3162-3p and hsa-miR-890 were downregulated in HSCs activated by TGF-*β*1 treatment. Similarly, previous studies have shown that miR-146 was profibrogenic, while miR-30 was antifibrogenic (Chen et al., 2017; Ge et al., 2016). However, there are also some conflicting results with former studies. Chen et al. found that miR-150-5p expression in HSCs was downregulated after treatment with TGF-*β*1 (Chen et al., 2020b). However, in Chen’s study, HSCs were incubated with 10 ng/ml TGF-*β*1 for 48 h, while in our study, HSCs were incubated with 5 ng/ml TGF-*β*1 for 24 h, which might account for the inconsistent results. In addition, Caviglia et al. found that pan-deletion of miRNAs in HSCs had a negligible effect on HSC activation and liver fibrosis (Caviglia et al., 2018), which might be explained by the knockout of both profibrogenic and antifibrogenic miRNAs.

There are still several limitations in the present study. The number of samples from GSE59492 and GSE14323 was not large enough, inevitably including some bias during analysis. The transcriptome data being analyzed were from liver biopsies, the differential expression of these miRNAs in cirrhosis *versus* healthy liver might be accounted for by differential expression in hepatocytes, HSCs, endothelial cells, and Kupffer cells, etc., or through more complex interplays within liver tissues, which could be part of the reason why we did not detect differential expression of all identified miRNAs in LX2 cells. Although HSCs play a central role in the occurrence of liver cirrhosis, the identified miRNAs expression in other hepatic cells and their functions in the pathogenesis of liver cirrhosis deserve further elucidation.

## Conclusions

Based on bioinformatics analysis and cell study verification, we identified 13 differentially expressed miRNAs as potential biomarkers of liver cirrhosis. Moreover, we identified 361 regulatory pairs of miRNA-mRNA and 20 hub genes in liver cirrhosis, most of which were involved in collagen and ECM components, immune response, and cell adhesion. These results would provide novel mechanistic insights into the pathogenesis of liver cirrhosis and identify candidate targets for its treatment.

##  Supplemental Information

10.7717/peerj.11910/supp-1 Supplemental Information 1 Raw data of *in vitro* experiments

10.7717/peerj.11910/supp-2 Supplemental Information 2 Uncropped Gels of Fig. 8

## Additional Information and Declarations

### Data Availability

The following information was supplied regarding data availability: The raw data of *in vitro* experiments are available in the Supplemental Files.
