# Supplementary figures and images for: Identification of miRNA-target gene regulatory networks in liver fibrosis based on bioinformatics analysis
Source: PeerJ. 2021 Aug 6;9:e11910. doi: 10.7717/peerj.11910 (PMC8351572; doi:10.7717/peerj.11910)

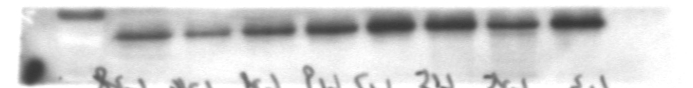

Supplement: Supplemental Information 2 [file peerj-09-11910-s002.zip › Fig. 8B WB gels/a-SMA.jpg]

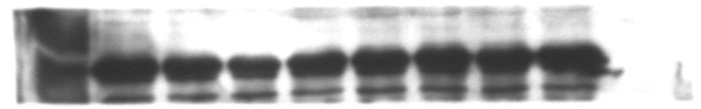

Supplement: Supplemental Information 2 [file peerj-09-11910-s002.zip › Fig. 8B WB gels/GAPDH.jpg]
